# Colorectal cancer screening test preferences by sociodemographic factors and health beliefs in diverse underserved populations

**DOI:** 10.21203/rs.3.rs-8652064/v1

**Published:** 2026-01-29

**Authors:** Aasma Shaukat, Taranika Sarkar Das, Jahnavi Udaikumar, Xucong Meng, Mahnoor Khan, Ayesha Nasir, Sarah Miller, Mark Pochapin

**Affiliations:** New York University Langone Medical Center; New York University Langone Medical Center; New York University Langone Medical Center; New York University Langone Medical Center; Mount Sinai Medical Center; New York University Langone Medical Center; Mount Sinai Medical Center; New York University Langone Medical Center

## Abstract

**Background:**

Despite the availability of multiple screening options, rates of colorectal cancer (CRC) screening remain suboptimal. With recent approval of a blood test for CRC screening, there is an urgent need to understand screening preferences of populations with low screening rates.

**Methods:**

Between October 2023 and June 2024, we conducted a survey among adults aged 45–75 at ambulatory primary care clinics across multiple community health centers and federally qualified healthcare centers across the city as well as in community settings regardless of prior screening

**Results:**

A total of 1,014 individuals completed the survey. Respondents were 12.8% Black/African American, 51.6% White, 23.4% Hispanic, 15.8% South Asian, and 4.2% Asian. Overall, the highest test preference was for screening colonoscopy (45.5%) followed by blood test (29.9%). Colonoscopy was preferred by individuals under age 70 (47.5%), while stool-based (20.2%) and blood-based (31.9%) tests were the most preferred among above 70 years (p = 0.0429. Whites (54.6%), Blacks (44.6%), and Hispanics (35.9%, p < 0.001) preferred colonoscopy, while Asians (37.2%) and South Asians (24.4%) favored blood tests. Factors associated with preference for a colonoscopy over other screening tests were younger age: respondents aged below 70 years were more likely to prefer colonoscopy, compared to respondents aged above 70 years (OR = 1.72, 95% CI [1.20–2.47], p = 0.003); Nonsmoker compared to former/current smokers (OR = 2.04, 95% CI: [1.10–3.94], p = 0.028); Having undergone a prior colonoscopy (OR = 6.83, 95% CI: [4.52–10.6], p = < 0.001) or not having a prior stool test (OR = 1.56, 95% CI: [1.52–2.11], p = < 0.001). Factors associated with preference for a blood test over other screening tests were education level: respondents without any college experience were more likely to prefer blood test compared to respondents with college experience (OR = 1.46, 95% CI: 1.02–2.07, p = 0.038); Nonsmoker compared to former/current smokers (OR = 1.73, 95% CI: [1.00–2.99], p = 0.048); Never undergone a prior colonoscopy (OR = 1.76, 95% CI: [1.23–2.51], p = 0.002). Factors associated with preference for a stool test over other screening tests were: age over 80 years compared to respondents aged below 80 (OR = 3.34, 95% CI: 1.67–6.55, p < 0.001); respondents with college experience were more likely to prefer blood test compared to respondents without college experience (OR = 1.62, 95% CI: 1.02–2.66, p = 0.048);

**Conclusion:**

The study underscores importance of patient preference in deciding which tests to offer. Tailored strategies addressing demographic, socioeconomic, and health belief barriers are needed to improve CRC screening adherence.

## Introduction

Screening for colorectal cancer (CRC) is effective at reducing the incidence of, and mortality from CRC.^1–3^ In the US, there are multiple CRC screening options available.^4^ Despite the availability of multiple screening options, rates of CRC screening remain suboptimal.^5^ With the inclusion of 45–49 year olds into the screening pool, current CRC screening rates are at 59%.^5, 6^ Screening rates are notably lower in populations with high burden of social determinants of health, such as federally qualified healthcare centers.^7^ Barriers to uptake of screening include lack of health insurance, lack of provider recommendations, diverse health beliefs and health literacy levels.^8^

In 2024, a blood test was approved for CRC screening.^9^ Most recently, the test was added to the National Comprehensive Cancer Network CRC screening guidelines.^10^ It is unknown whether the option of a blood test for CRC screening will drive the unscreened individuals to complete scree^7^ning, especially in underserved populations.

In a survey study of CRC screening preferences among 1000 unscreened Americans, Makaroff et al. presented individuals with the 5 options in the US multi society task force guidelines, and also asked participants to choose between annual fecal immunochemical test or screening colonoscopy.^11^ Majority of the participants selected noninvasive option of stool tests as their preferred option, regardless of their age. However, the survey did not include blood test as an option for CRC screening, and it is not known how many individuals included were from underserved populations. Given the continually changing racial and ethnic demographics of the screening-eligible population and recent inclusion of those aged 45–50 years by US guidelines^4, 12, 13^, it is important to understand the preferences of CRC screening tests by race, ethnicity, social and demographic factors in diverse patient populations, many of whom have the lowest rates of screening in the US.^14^

The aim of our study was to understand the screening preferences and the demographic, social and cognitive factors influencing the choice of one method over another in multiple diverse underserved populations through a multi-institutional collaboration. The survey study also aimed to understand the perspectives on various tests by race/ethnicity and sociodemographic factors to better guide outreach efforts and policy development.

## Methods

Between October 2023 and June 2024, an anonymous survey was administered to adults aged 45–75 who could read or speak English or Spanish. A multi-pronged method was used to recruit individuals to participate in the research study. Our recruitment methods included: (1) Primary care patients across our healthcare systems were emailed the survey in their preferred language, regardless of prior screening or lack of screening; (2) On-site surveys were administered at multiple primary care clinics and community settings across the city, located in predominantly non-white population neighborhoods, or serving the unsured and underserved populations, such as federally qualified healthcare centers in Brooklyn. An on-site coordinator screened patients in the clinics’ waiting room and post-visit, to ensure they met the age criteria and had not yet completed the survey; (3) Surveys were also administered during community meetings and in shared public spaces (e.g., hospital lobby). In-person surveys were completed either on an iPad provided by the study team, or with pencil and paper, or through a QR code that participants could scan and complete using their personal cell phones. Participants were offered a $5 gift card for completing the survey. Participation was voluntary.

The study was conducted at NYU Brooklyn primary care clinics, NYU Family Health Centers (federally qualified health care centers), Bellevue Hospital primary care clinic, NYU Manhattan primary care clinics, external sites at the primary care clinics associated with Mount Sinai in Manhattan, Mount Sinai South Nassau, a community clinic in Queens, and in community settings (e.g., community meetings, shared spaces). The survey was approved by the IRB of each institution.

Respondents were given information about the different CRC screening options of colonoscopy, stool test and a blood test, and asked questions about their relative preference and all applicable reasons for their choice over other tests. We developed a survey conveying basic information about the performance of colonoscopy, stool test and blood test, with pros and cons, using information from public websites such as CDC^15^, and recent publications on blood tests.^9^ We also included one question about their view on the idea of future availability of a multi-cancer detection test that could screen for multiple cancers, without providing specific performance data. The survey was field tested by a community engagement committee at NYU Langone, and underwent review and edits by the health literacy committee for NYU Family health centers for readability and comprehension to a 2nd grade reading level. Certified Spanish translation was obtained per IRB requirements.

### Statistical Analysis:

The primary outcome was test preference of the participants. Baseline analyses included chi-square tests for categorical variables and t-tests for continuous variables to calculate p-values. Three multivariable logistic models were created comparing colonoscopy, blood test and stool test to others respectively: one comparing blood tests to other screening methods and another comparing blood or stool tests to colonoscopy. Univariate analysis and LASSO regression were performed to identify suitable covariates. For the model comparing blood tests to other screening methods, the covariates included age, education, smoking status, and prior colonoscopy. For the model comparing blood and stool tests to colonoscopy, the covariates included age, smoking status, health insurance, prior colonoscopy, and prior stool-based tests. Percentages of responses to reasoning questions were calculated to determine the most and least common reasons for patients’ choices. Since some patients did not answer all questions, the models were based on complete cases for both the primary outcome and the covariates.

## Results

### Overall results:

**Figure 1:**
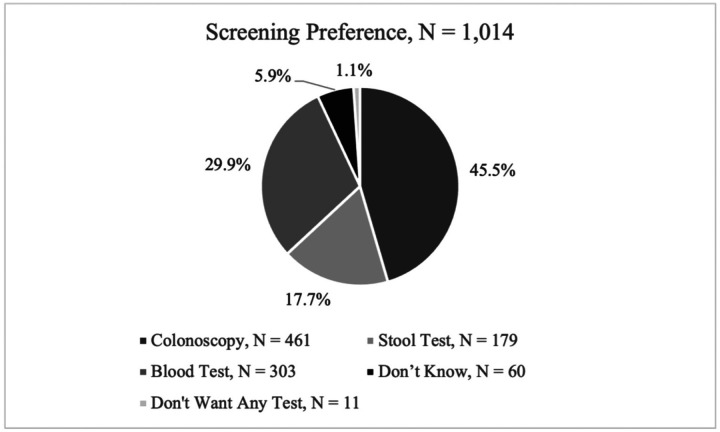
Overall reported screening preference

A total of 1,014 individuals completed the survey. Most respondents were aged 65–70 (19%) and 39.8% were male, 12.8% Black/African American, 51.6% White, 23.4% Hispanic, 15.8% South Asian, and 4.2% Asian. (Table 1) Overall, the highest test preference was for screening colonoscopy (45.5%) followed by blood test (29.9%). 71 participants (7.0%) did not indicate a preference for any modality (Figure 1). Women showed a slightly higher preference for colonoscopy compared to men (46.1% vs. 44.3% respectively; p = 0.0171).

### Preferences by sociodemographic factors:

Screening preferences varied by age: colonoscopy was preferred by individuals under age 70 (47.5%), while stool-based (20.2%) and blood-based (31.9%) tests were the most preferred among above 70 years (p = 0.0429). For every 5-year increase in age from 45 to 80 years, the odds of favoring colonoscopy decreased by 8.4% (p = 0.014). CRC screening preferences varied significantly by race (p < 0.0001): Whites (54.6%), Blacks (44.6%), and Hispanics (35.9%, p <0.001) preferred colonoscopy, while Asians (37.2%) and South Asians (24.4%) favored blood tests. Colonoscopy was the preferred option for college-educated individuals (53.1%), those that were employed (45.2%) and individuals with health insurance (48.6%). Respondents who perceived themselves at a higher risk for colon cancer were more likely to prefer colonoscopy (64.4% for those with “large” perceived risk and 72.4% for “very large” risk) compared to other screening tests (p < 0.0001). Non-smokers preferred colonoscopy (47.4%), while smokers preferred blood tests (45.2%, p = 0.090). Prior use reinforced choices: 57.3% of prior colonoscopy users and 37.3% of prior stool test users reported preference for the same modality of screening as prior (p < 0.0001).

### Barriers and facilitators for each screening modality:

Participants who preferred a screening modality were asked to cite reason(s)s for their selection. Results are given by modality below.

### Colonoscopy:

Among participants who selected colonoscopy as the preferred option (n=461, 45.5%) citing the following reasons and beliefs: 54.7% chose colonoscopy based on their doctor’s advice, 53.6% valued its capability to detect polyps and cancer, and 18.0% appreciated the 10-year interval between screenings. For those that did not select colonoscopy as the preferred option (n=553, 54.5%), reasons cited were: Concerns for discomfort with the bowel preparation (46.2%), stress associated with the test (20.8%), and logistical issues such as time off work (11.3%).

### Stool Test:

For those that selected stool test as the preferred option (n=179; 17.7%), reasons cited were: Simplicity (69.0%), 46.8% preferred the ability to complete the test at home; 45.61% appreciated not needing bowel preparation, 12.3% favored stool tests for being cost-effective compared to colonoscopy.

### Blood Test:

Participants who selected blood test as the preferred option (N=303; 29.9%), cited the following reasons: Ease and Comfort of the test (82.7%), convenience during routine visits (56.6%), no need for stool sample submission (36.2%).

### Predictors of preference for colonoscopy:

From multivariate model, factors associated with preference for a colonoscopy over other screening tests were younger age: respondents aged below 70 years were more likely to prefer colonoscopy, compared to respondents aged above 70 years (OR = 1.72, 95% CI [1.20 – 2.47], p = 0.003); Nonsmoker compared to former/current smokers (OR = 2.04, 95% CI: [1.10 – 3.94], p = 0.028); Having undergone a prior colonoscopy (OR = 6.83, 95% CI: [4.52 – 10.6], p = <0.001) or not having a prior stool test (OR = 1.56, 95% CI: [1.52 – 2.11], p = <0.001)

### Predictors of preference for blood test:

Factors associated with preference for a blood test over other screening tests were education level: respondents without any college experience were more likely to prefer blood test compared to respondents with college experience (OR = 1.46, 95% CI: 1.02–2.07, p = 0.038); Nonsmoker compared to former/current smokers (OR = 1.73, 95% CI: [1.00 – 2.99], p = 0.048); Never undergone a prior colonoscopy (OR = 1.76, 95% CI: [1.23 – 2.51], p = 0.002)

### Predictors of preference for stool test:

Factors associated with preference for a stool test over other screening tests were: age over 80 years compared to respondents aged below 80 (OR = 3.34, 95% CI: 1.67–6.55, p < 0.001); respondents with college experience were more likely to prefer blood test compared to respondents without college experience (OR = 1.62, 95% CI: 1.02–2.66, p = 0.048); Respondents have seen less than 15 physicians last year compared to those who have seen greater than 15 physicians (OR = 3.26, 95% CI: 1.44–8.83, p = 0.010); Not having health insurance compared have a health insurance (OR = 2.36, 95% CI: 1.27–4.37, p = 0.0); Never undergone a prior colonoscopy (OR = 3.29, 95% CI: [1.27–4.37], p = <0.001) or having a prior stool test (OR = 2.08, 95% CI: [1.40–3.11], p = <0.001)

### Multi cancer blood test compared to current test for colon cancer:

**Figure 3:**
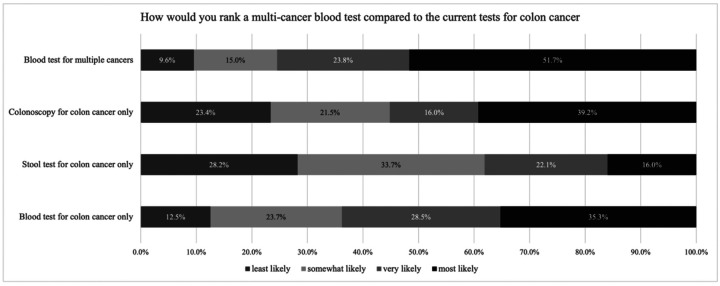
Likelihood of Completing Multi-Cancer Blood Tests Compared to Other CRC Screening Methods

When asked about a future multi-cancer blood test, participants indicated the highest interest in completing a multi-cancer blood (51.7%), compared to 39.2% for colonoscopy, 25.3% for colon cancer-specific blood tests, and 16.0% for stool tests. (Figure 3)

## Discussion

In a multi-site survey of diverse, underserved populations across New York City we found the highest preference for screening test to be for colonoscopy followed by blood test. Preference was higher for blood test among older individuals, those with no education, health insurance and smokers, suggesting the perceived simplicity of completing a blood test being valued among these participants. Participants who perceived themselves at increased risk for CRC preferred colonoscopy. Of note, we did not ask about family history, and individuals with family history may perceive themselves at increased risk of CRC, where screening colonoscopy is appropriate. Participants with prior history of screening preferred the same screening modality, suggesting familiarity and comfort with the modality. Similar to other studies, we found that physician recommendation was heavily valued by participants in selecting a test.^16^ Our findings differ from those by Makaroff et al, in that we found the highest preference for colonoscopy among participants^17^ but similar to findings by others for finding preference for same screening modality for those with prior screening.^18^

Despite advances in CRC screening, significant disparities persist among racial and ethnic groups. National and statewide health surveys indicate that screening rates remain low among Hispanic Americans and suggest a widening gap in screening rates among Black and American Indian/Alaskan Native populations compared to non-Hispanic Whites.^19, 20^ With the availability of blood test for CRC screening, it is important for healthcare systems to assess which choices they should offer for their patient populations and resources. Multiple studies and microsimulation^21, 22^ have reported that compared to FIT and colonoscopy, screening blood test results in less mortality reduction and increased costs but, compared to no screening, offers better quality-adjusted life years (QALY) per 1,000 individuals and is more cost-effective. The studies rely on many assumptions regarding adherence to existing and new modalities, but the real world adherence to initial and follow up blood based testing is not known. Our study provides context of level of interest in diverse and underserved populations for blood test. We did not ask about repeat testing, as the repeat interval for blood tests is currently unknown, but proposed to be triennial. Comparison of annual vs triennial testing for stool and blood tests should be conducted in future studies, along with benefit on long term outcomes such as CRC incidence and mortality.

Our study has several limitations: We did not capture perspectives from American Indian and Alaska Native populations. We were unable to include individuals with limited reading and writing abilities, or those who read or speak languages other than Spanish or English. Data on variables of interest, such as smoking or prior screening is by self-report and not verified through the electronic health record, although previous research has shown that self-report of CRC screening is generally accurate.^23^ And while we sampled a diverse population, the generalizability of our findings is unknown. It is likely that individuals more interested in the topic or more comfortable discussing CRC screening were more likely to complete the survey.^23 24^ Other limitations are that preference may not translate into what participants choose to do or not do, and we do not know the level of adherence to initial or repeat testing in these participants.

Despite its limitations, this study is the largest of its kind to examine patients’ perspectives on screening tests and provide insights into their socioeconomic demographics. It focuses on multiple race/ethnicity groups across multiple healthcare systems many of whom are underserved, and screening rates have consistently been low. The study provides valuable insights for clinics with diverse patient populations, in approaching the tests their population is most likely to complete. The study also demonstrates the need for patient level education and awareness on the pros and cons of CRC screening engagement to garner engagement and adherence.

## Figures and Tables

**Figure 2:**
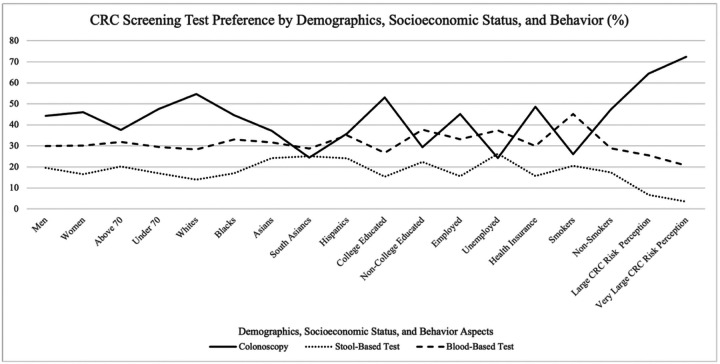
Preference of CRC screening test by sociodemographic factors

**Table 1.** Demographics of participants, and by Test Choice

Characteristic	Colonoscopy, N = 461	Stool Test, N = 179	Blood Test, N = 303	Don’t Know, N = 60	Don’t want to choose, N = 11	Overall, N = 1014
**Gender**						
Men	179 (44.3%)	79 (19.6%)	121 (30.0%)	21 (5.2%)	4 (1.0%)	404 (40.2%)
Women	275 (46.1%)	99 (16.6%)	180 (30.2%)	36 (6.0%)	7 (1.2%)	597 (59.4%)
Transgender	0 (0.0%)	0 (0.0%)	1 (100.0%)	0 (0.0%)	0 (0.0%)	1 (0.1%)
Nonbinary	1 (33.3%)	0 (0.0%)	0 (0.0%)	2 (66.7%)	0 (0.0%)	3 (0.3%)
**Age**						
above 70	80 (37.6%)	43 (20.2%)	68 (31.9%)	18 (8.5%)	213 (21.17%)	213 (21.2%)
below 70	377 (47.5%)	135 (17.0%)	234 (29.5%)	40 (5.0%)	793 (78.83%)	793 (78.8%)
**Race**						
Black / African American	58 (44.6%)	22 (16.9%)	43 (33.1%)	5 (3.8%)	2 (1.5%)	130 (13.1%)
White	286 (54.7%)	73 (14.0%)	148 (28.3%)	14 (2.7%)	2 (0.4%)	523 (52.5%)
American Indian / Alaska Native	1 (100.0%)	0 (0.0%)	0 (0.0%)	0 (0.0%)	0 (0.0%)	1 (0.1%)
Asian (Except India)	16 (37.2%)	7 (16.3%)	17 (39.5%)	3 (7.0%)	0 (0.0%)	43 (4.3%)
Native Hawaiian or Other Pacific Islander	2 (66.7%)	0 (0.0%)	0 (0.0%)	1 (33.3%)	0 (0.0%)	3 (0.3%)
Other	54 (39.7%)	33 (24.3%)	43 (31.6%)	5 (3.7%)	1 (0.7%)	136 (13.7%)
India	39 (24.4%)	41 (25.6%)	46 (28.8%)	28 (17.5%)	6 (3.8%)	160 (16.1%)
**Hispanic**	85 (35.9%)	57 (24.1%)	83 (35.0%)	11 (4.6%)	1 (0.4%)	237 (23.8%)
**Education**						
Any college	356 (53.1%)	103 (15.4%)	179 (26.7%)	27 (4.0%)	6 (0.9%)	671 (68.3%)
No college	91 (29.2%)	70 (22.4%)	118 (37.8%)	28 (9.0%)	5 (1.6%)	312 (31.7%)
**Profession**						
Employee	136 (45.2%)	47 (15.6%)	100 (33.2%)	16 (5.3%)	2 (0.7%)	301 (30.2%)
Mangement	45 (65.2%)	10 (14.5%)	13 (18.8%)	1 (1.4%)	0 (0.0%)	69 (6.9%)
Self Employed	57 (55.9%)	20 (19.6%)	21 (20.6%)	3 (2.9%)	1 (1.0%)	102 (10.2%)
Homemaker	24 (33.8%)	14 (19.7%)	23 (32.4%)	7 (9.9%)	3 (4.2%)	71 (7.1%)
Unemployed	22 (24.2%)	24 (26.4%)	34 (37.4%)	10 (11.0%)	1 (1.1%)	91 (9.1%)
Retired	146 (46.8%)	54 (17.3%)	95 (30.4%)	13 (4.2%)	4 (1.3%)	312 (31.3%)
On Disability	1 (25.0%)	1 (25.0%)	1 (25.0%)	1 (25.0%)	0 (0.0%)	4 (0.4%)
Multiple Professions	24 (51.1%)	7 (14.9%)	16 (34.0%)	0 (0.0%)	0 (0.0%)	47 (4.7%)
**Overall Health**						
Very Poor	5 (45.5%)	3 (27.3%)	3 (27.3%)	0 (0.0%)	0 (0.0%)	11 (1.09%)
Poor	50 (34.5%)	31 (21.4%)	54 (37.2%)	9 (6.2%)	1 (0.7%)	145 (14.40%)
Good	309 (47.0%)	112 (17.0%)	193 (29.4%)	35 (5.3%)	8 (1.2%)	657 (65.24%)
Very Good	97 (50.0%)	33 (17.0%)	51 (26.3%)	11 (5.7%)	2 (1.0%)	194 (19.27%)
**CRC Risk**						
Very Small	64 (38.3%)	46 (27.5%)	46 (27.5%)	9 (5.4%)	2 (1.2%)	167 (16.70%)
Small	236 (45.8%)	89 (17.3%)	166 (32.2%)	21 (4.1%)	3 (0.6%)	515 (51.50%)
Large	96 (64.4%)	10 (6.7%)	38 (25.5%)	5 (3.4%)	0 (0.0%)	149 (14.90%)
Very Large	21 (72.4%)	1 (3.4%)	6 (20.7%)	1 (3.4%)	0 (0.0%)	29 (2.90%)
Not at risk	43 (30.7%)	31 (22.1%)	45 (32.1%)	15 (10.7%)	6 (4.3%)	140 (14.00%)
**Smoking**						
Not smoker	442 (47.4%)	162 (17.4%)	269 (28.8%)	50 (5.4%)	10 (1.1%)	933 (92.74%)
Smoker	19 (26.0%)	15 (20.5%)	33 (45.2%)	5 (6.8%)	1 (1.4%)	73 (7.26%)
**Number of Physicians**						
Above 15	37 (44.0%)	8 (9.5%)	33 (39.3%)	5 (6.0%)	1 (1.2%)	84 (8.39%)
Below 15	405 (47.1%)	154 (17.9%)	254 (29.6%)	39 (4.5%)	7 (0.8%)	859 (85.81%)
I don’t know	19 (32.8%)	15 (25.9%)	14 (24.1%)	7 (12.1%)	3 (5.2%)	58 (5.79%)
**Health Insurance**	436 (48.6%)	141 (15.7%)	268 (29.9%)	44 (4.9%)	8 (0.9%)	897 (89.97%)
**Priorcrcscreening**	381 (51.4%)	119 (16.1%)	220 (29.7%)	20 (2.7%)	1 (0.1%)	741 (78.33%)
**Priorcolo**	401 (57.3%)	83 (11.9%)	191 (27.3%)	22 (3.1%)	3 (0.4%)	700 (71.36%)
**Stoolbasedtest**	179 (37.3%)	119 (24.8%)	153 (31.9%)	23 (4.8%)	6 (1.3%)	480 (48.63%)

**Table 2:** Reasons for test preference

Reasoning for Test Preference	Count	Percentage
Stool Test		
1. Why did you choose a stool test?	174	
The simplicity of the stool test	120	69.0%
The stool test was recommended to me	54	31.0%
A positive stool test does not necessarily mean I have cancer	8	4.6%
The stool test is cheaper than a colonoscopy	17	9.8%
I don’t know	9	5.2%
I can complete it at home and mail it in	28	16.1%
		
2. In your opinion what are the advantages of the stool test compared to the colonoscopy for you?	171	
I did not feel comfortable with the colonoscopy	43	25.1%
I can complete the test at home	80	46.8%
The test is less painful	45	26.3%
I don’t have to go through the bowel preparation	78	45.6%
I don’t have to travel or take a day off work	52	30.4%
I don’t know any gastroenterologist	11	6.4%
I can discuss the result with my doctor	14	8.2%
I trust in measured values	7	4.1%
A stool test is less stressful	67	39.2%
The stool test is cheaper than a colonoscopy	21	12.3%
My doctor did not recommend it to me	14	8.2%
I don’t know	7	4.1%
Other: I prefer a lab test	3	1.8%
3. In your opinion what are the advantages of the stool test compared to the blood test for you?	167	
I feel uncomfortable with having a blood draw	19	11.4%
I am scared of the blood draw	7	4.2%
A stool test is more accurate	39	23.4%
I trust a stool test more than a blood test	26	15.6%
I am used to submitting stool tests for CRC screening	39	23.4%
I don’t know	60	35.9%
		
Colonoscopy		
For those who preferred colonoscopy		
1. What are the advantages of colonoscopy over blood or stool tests for you?	455	
My doctor recommended it	249	54.7%
It’s the best test for colon cancer screening	279	61.3%
It’s due every 10 years	82	18.0%
It looks for polyps and cancer	244	53.6%
My friends or family member had one and recommended it	43	9.5%
		
For those who didn’t prefer colonoscopy		
1. Why did you decide not to choose to do a colonoscopy?	485	
I am not comfortable with the test or with the subject ‘colon cancer’	54	11.1%
I am not comfortable with the preparation necessary for colonoscopy	224	46.2%
I am at low risk for colon cancer	52	10.7%
I am healthy and do not need to be screened	21	4.3%
I have not thought about this topic	41	8.5%
I don’t have time for the preparation and the colonoscopy	55	11.3%
I am scared of a cancer diagnosis	17	3.5%
I believe that colonoscopy is painful	38	7.8%
I believe that colonoscopy will cause physical stress	39	8.0%
The idea of colonoscopy is stressful	101	20.8%
My physician did not recommend colonoscopy	41	8.5%
Friends advised against colonoscopy	1	0.2%
I am unable to be away from work for the procedure	32	6.6%
I don’t know	62	12.8%
		
Blood test		
1. Why did you choose a blood test?	295	
The ease / comfort of a blood test	244	82.7%
The convenience of giving blood at the doctor’s office at the same visit	167	56.6%
A positive blood test result does not necessarily mean that I have colon cancer	15	5.1%
The blood test is cheaper than a colonoscopy	36	12.2%
I don’t know	10	3.4%
The blood test was recommended to me	1	0.3%
		
2. What are the advantages of the blood test compared to the stool test for you? Select all that apply	290	
I don’t feel comfortable with the submission of a stool sample	76	26.2%
I don’t have to collect the sample myself. It is the responsibility of the physician/laboratory	105	36.2%
I don’t need to store stool samples in my refrigerator	73	25.2%
I could complete my test in one visit	144	49.7%
I trust a blood test more than a stool test	69	23.8%
My toilet is not suitable for taking a stool sample	6	2.1%
I don’t know	30	10.3%
		
For those who didn’t choose any test		
1. What would you need to change to convince you to take one of the tests?	45	
The test must have no out-of-pocket cost	5	11.1%
The test should be recommended to me by my doctor	10	22.2%
I would need to know more about the test	11	24.4%
I don’t know	19	42.2%

## Data Availability

Deidentified data will be available upon request and approvals
